# Imatinib-induced rhabdomyolysis: A case report

**DOI:** 10.1016/j.lrr.2026.100561

**Published:** 2026-01-12

**Authors:** Jordan Rubenstein, Sarah M. Schwartz, Jay Vankawala, Michael Caplan, Steven Shea, Joseph G. Jurcic

**Affiliations:** Columbia University Vagelos College of Physicians and Surgeons, New York, NY, the United States of America

**Keywords:** Imatinib, Toxicity, Tyrosine kinase inhibitor, Chronic myeloid leukemia, Rhabdomyolysis

## Abstract

Imatinib, a tyrosine kinase inhibitor used for chronic myeloid leukemia (CML) and gastrointestinal stromal tumors (GIST), is associated with myalgia and creatine kinase (CK) elevations, though severe rhabdomyolysis and myopathy are rare. We report an 83-year-old woman with CML who developed progressive proximal weakness, dark urine, and acute kidney injury after three years of imatinib therapy. Laboratory evaluation revealed CK >24,000 U/L, transaminitis, and myoglobinuria. MRI showed diffuse muscle and fascial edema, while autoimmune testing was negative. Imatinib and rosuvastatin were discontinued, and the patient was managed with intravenous fluids and supportive care. CK and renal function normalized within 10 days, with substantial recovery of strength. The strong temporal relationship between drug withdrawal and improvement implicates imatinib as the etiology. This case represents one of the most severe reported instances of imatinib-induced rhabdomyolysis. Early recognition and discontinuation are essential to prevent life-threatening sequelae.

## 1 Introduction

Myalgia is a commonly reported side effect in patients taking imatinib for either chronic myeloid leukemia (CML) or gastrointestinal stromal tumor (GIST) [1]. Although the manufacturer’s labeling only reports creatine kinase (CK) elevations in 1–10% of patients [2], postmarketing analysis has shown CK abnormalities in over 50% of fifty patients with CML treated with imatinib over the course of a year [3]. Even more atypical are cases of muscle edema [4] and rhabdomyolysis [5], which are rarely reported. Below we describe a case of rhabdomyolysis and myositis in the setting of imatinib toxicity.

## 2 Case report

We present a case of an 83-year-old woman with an 11-year history of CML who presented to an outside hospital with four months of progressive muscle weakness and dark urine. With regard to her CML, she was initially treated with dasatinib but was switched to imatinib 200 mg daily three years prior to admission because of the development of bilateral pleural effusions. She had a sustained deep molecular response (MR 4.5) since that time. Most recently PCR testing one month prior to admission showed no detectable BCR-ABL transcripts. She presented to an outside hospital with four months of progressive muscle weakness and dark urine. At baseline, the patient was able to ambulate with a walker and her progressive inability to do so over the preceding months prompted her presentation. She was found to have elevated creatinine kinase, myoglobinuria, and an acute kidney injury with creatinine elevated to 4.90 mg/dL from baseline of 0.75 mg/dL concerning for rhabdomyolysis. She underwent thigh MRI that showed diffuse muscle edema, diffuse subcutaneous edema, and edema along some fascial planes. She was then transferred to our institution for further evaluation.

She otherwise has a medical history of hyperlipidemia on rosuvastatin 40 mg daily. On arrival, her physical exam was notable for intact 5/5 strength on wrist flexion/extension, grip, dorsiflexion, and plantarflexion, with 3/5 strength on shoulder abduction, bicep and triceps flexion and 2/5 strength on hip flexion. She was also noted to have 2+ pitting edema in her bilateral lower extremities, and diffuse erythema and bruising without rash on both upper and lower extremities, with unremarkable cardiopulmonary exam.

Blood tests were notable for elevated creatine kinase >24,000 U/L, AST 1,152 U/L, and ALT 917 U/L (Table 1). Given her physical exam with symmetrical and proximal weakness, electromyography (EMG) was deferred as a neurologic etiology was thought to be less likely. In demyelinating disease, one would expect a patchier, non-symmetrical pattern of pathology and with axonal degeneration, distal involvement would be expected first. Serologic studies for a rheumatologic etiology of her weakness, including polymyositis and dermatomyositis, were negative (Table 1).

**Table 1:** Serological testing.

**Parameter**	**Result**	**Reference Range**
Hemoglobin	11.1 g/dL	11.2–14.7 g/dL
Erythrocyte Sedimentation Rate	22 mm/h	0–30 mm/h
**Creatinine**	**4.90mg/dL**	**0.50–0.95mg/dL**
**Aspartate Aminotransferase**	**1152U/L**	**10–37U/L**
**Alanine Aminotransferase**	**917U/L**	**≤ 34U/L**
Lactate Dehydrogenase	261 U/L	120–250 U/L
**Creatine Kinase**	**>24,000U/L**	**26–192U/L**
Troponin 1	0.240 ng/mL	<0.04 ng/mL
ANA	Negative	Negative
Connective Tissue Disorders panel: *Centromere, SCL, KU, PM-SCL, Smith-RNP*	Negative	Negative
Lupus Panel: *dsDNA, smith*	Negative	Negative
Poly- and Dermatomyositis Panel: *EJ, Jo1, MI-2a, OJ, PL-12, PL-7, SRP*	Negative	Negative
Sjogren’s Panel negative: *RO/LA*	Negative	Negative
Myasthenia Gravis Panel: *Musk, acetylcholine receptor*	Negative	Negative
Vasculites Panel: *Myeloperoxidase, ANCA, CANCA*	Negative	Negative
Other Serological Markers: *SAE, Fibrillarin, NXP, MDA5, TIF-1, P155/140, HMGCR Ab*	Negative	Negative

The bold values are those that are high compared to the reference values and are clinically relevant.

No immunosuppressive or glucocorticoid therapy was started. She was given intravenous fluids for her acute kidney injury secondary to rhabdomyolysis. Imatinib was discontinued given suspicion for contribution to the clinical picture. It was recommended that she remain off CML-directed therapy with frequent PCR monitoring for BCR-ABL transcripts since she has a documented deep molecular response for more than two years. Rosuvastatin was also held. Approximately ten days after stopping imatinib, the patient had normalization of CK, and Cr, with improvement in muscle strength. On her last examination prior to discharge, strength grading of shoulder abduction, bicep and triceps flexion, and hip flexion had improved to 4-5/5. She became more independent and mobile in bed and was able to eat by herself. Since no benefit was expected from immunosuppressive therapy, physical therapy and rehabilitation were prescribed with close oncologic follow-up. Ultimately, the strict time association between improvement and treatment suspension in the absence of other identifiable triggers highly suggests the diagnosis of imatinib-induced rhabdomyolysis.

## 3 Discussion

Few cases of rhabdomyolysis induced by imatinib are described in the literature; however, most of these cases report moderate creatine kinase elevations of ∼200–1000 U/L (reference range 30–135 U/L) [4]. To the best of our knowledge, this is one of the first cases with substantial creatine kinase elevations >24,000 U/L. Similar to other cases, the proximal muscle weakness and MRI findings suggest myositis and the absence of any key serological markers strongly refutes a rheumatologic etiology in the case of this patient [10].

Rhabdomyolysis is a clinical syndrome that results from rapid breakdown of damaged skeletal muscle, leading to the release of their intracellular contents – primarily creatine kinase, myoglobin, and electrolytes – into the bloodstream. The condition can be triggered by a variety of insults, including trauma, prolonged immobilization, medications, toxins, and metabolic disturbances. While CK is a sensitive biomarker of muscle injury, myoglobinuria, though classic, is not always present and is not required for diagnosis. The clinical presentation can range from asymptomatic elevation of muscle enzymes to life-threatening acute kidney injury. Identification of the underlying cause is crucial, as certain etiologies, such as drug-induced or idiopathic inflammatory myopathy, may require disease-specific therapies beyond supportive care. Early recognition, prompt intravenous fluid administration, and removal or discontinuation of the offending agent remain the cornerstone of management, aiming to preserve renal function and prevent systemic complications [5].

The mechanism whereby imatinib may cause creatine kinase elevation is not well understood. Imatinib is a selective tyrosine kinase inhibitor targeting the Philadelphia chromosome (BCR-ABL fusion oncoprotein) in CML, however, the drug also has action at several other tyrosine kinases, including platelet derived growth factor receptor (PDGFR) and c-abl [6]. Both of these receptors are expressed by muscle tissue [7,8], and it is possible that inhibition of these enzymes by imatinib leads to this toxicity. It has been demonstrated that cardiomyocytes in culture, when treated with imatinib, showed a toxic response, with retroviral gene transfer of an imatinib-resistance mutant of c-abl attenuating this effect [9]. Perhaps a similar response occurs in skeletal muscle. It is thought that perhaps the commonly experienced myalgia side effects of imatinib, may represent an early effect of the drug on muscle [4].

## 4 Conclusion

We report a rare case of rhabdomyolysis and myositis associated with imatinib use in CML. The strong temporal relationship between onset and resolution of rhabdomyolysis and the start and discontinuation of imatinib strongly suggests a drug-induced etiology. Ultimately, this case underlines the importance of clinician awareness of this manifestation.

## CRediT authorship contribution statement

**Jordan Rubenstein:** Conceptualization, Formal analysis, Investigation, Writing – original draft, Writing – review & editing. **Sarah M. Schwartz:** Supervision, Writing – review & editing. **Jay Vankawala:** Supervision. **Michael Caplan:** Supervision. **Steven Shea:** Conceptualization, Supervision, Writing – review & editing. **Joseph G. Jurcic:** Conceptualization, Supervision, Writing – review & editing.

## Declaration of competing interest

The authors declare that they have no known competing financial interests or personal relationships that could have appeared to influence the work reported in this paper.
